# Proteome-wide Mendelian randomization and colocalization analysis identify CD5 as a plasma protein associated with cerebral palsy

**DOI:** 10.1097/MD.0000000000044693

**Published:** 2025-09-19

**Authors:** Chao Bai, Mingbo Hu, Hong Zhao, Jingxuan Xu, Junjie Wu, Xinping Luan, Song-hai Biedelehan

**Affiliations:** a Cerebral Palsy Center in Neurosurgery, The Second Affiliated Hospital of Xinjiang Medical University, Urumqi, Xinjiang, China.

**Keywords:** CD5, cerebral palsy, Mendelian randomization, plasma protein, proteomics

## Abstract

Cerebral palsy (CP), a neurodevelopmental disorder in children, remains incompletely understood, particularly regarding its etiology. The proteome offers potential therapeutic targets for a range of neurodevelopmental conditions. This investigation sought to explore the causal relationship between plasma proteins and CP risk through genome-wide Mendelian randomization (MR). Genetic instruments for 2923 plasma proteins were derived from extensive proteomic studies. Data on CP were obtained from publicly accessible datasets. Proteome-wide MR and colocalization analyses were employed to explore the causal impact of circulating proteins on CP. Protein–protein interaction and druggability assessments were performed to prioritize candidate therapeutic targets. Additionally, systematic MR analyses of healthy lifestyle factors and CP-associated proteins were executed to ascertain proteins that could serve as targets for intervention via lifestyle modifications. Genetically determined circulating levels of the plasma protein CD5 demonstrated significant associations with CP risk. Among the identified drug targets, baclofen has been used in the treatment of spastic CP, and CD5 levels can be modulated through healthy lifestyle interventions. This study identified CD5 as a circulating protein biomarker with strong causal evidence linking it to CP risk and highlighted it as a potential target for both pharmaceutical and lifestyle interventions, providing fresh perspectives on the mechanisms, prophylaxis, and management of CP.

## 1. Introduction

Cerebral palsy (CP) is a major cause of childhood disability and is widely recognized as a multifactorial disorder arising from genetic, prenatal, perinatal, and postnatal factors.^[1]^ Prenatal contributors include genetic variants, intrauterine infections, and maternal complications, whereas perinatal insults – particularly hypoxic–ischemic injury,^[2]^ prematurity,^[3]^ and low birth weight^[4]^ – are strongly associated with disease onset. Postnatal factors, such as severe neonatal infections and traumatic brain injury, may also increase the risk.^[5]^ Although its prevalence has remained relatively stable at 0.2% to 0.3% in recent decades, a modest increase has been observed, likely linked to the enhanced survival rates of premature infants resulting from advances in medical technologies.^[6]^

The etiological spectrum of CP is broad, and symptom presentation varies depending on the brain regions affected. Most cases are thought to originate during the perinatal period, with ischemic and hypoxic brain injury representing the predominant causes.^[7]^ Recent genetic studies have further suggested that hereditary factors contribute substantially to a subset of cases.^[8]^ In addition, evidence indicates that CP patients exhibit elevated levels of inflammatory proteins in cerebrospinal fluid and plasma compared with healthy children, suggesting that inflammatory responses – whether secondary to cerebral ischemia and hypoxia or idiopathic – play an important role in disease pathogenesis.^[9,10]^ Given this complexity, the identification of noninvasive early biomarkers is of great importance, both for improving our understanding of CP etiology and for advancing strategies in risk stratification, early intervention, and disease prevention.

As vital biomolecules that perform fundamental roles in organisms, circulating proteins serve as key indicators for understanding the overall health status of humans.^[11]^ Dysregulated protein expression or function has been associated with multiple pathological conditions, encompassing numerous neurological disorders, including Alzheimer’s disease, Parkinson’s disease, multiple sclerosis, and autism spectrum disorder.^[12,13]^ Epidemiological investigations have revealed associations between specific circulating protein levels and CP.^[14–16]^ However, these studies, along with many others, predominantly relied on observational designs involving limited protein types and quantities, which hindered the ability to fully clarify the link between circulating proteins and CP. Moreover, the potential for confounding biases and reverse causality in observational studies presents significant challenges for establishing causal relationships.

Recent comprehensive proteomics investigations have detected in excess of 18,000 protein quantitative trait loci encompassing more than 4000 proteins,^[17–19]^ providing a foundation for investigating the causal relationships between circulating proteins and human diseases through Mendelian randomization (MR) approaches. MR, an epidemiological method, enhances causal inference by utilizing genetic variants as instrumental variables (IVs) for exposures (e.g., circulating protein levels). This approach exhibits reduced susceptibility to reverse causation and confounding influences from unmeasured variables, including environmental or behavioral factors.^[20]^ In this investigation, a proteome-wide MR analysis was carried out to assess the causal effects of circulating proteins on CP risk. Following this, an evaluation was conducted to determine whether the discovered protein indicators could be influenced via pharmacological treatments or lifestyle interventions.

## 2. Materials and Methods

### 2.1. Research design

Two sample MR analyses were performed to investigate the associations between circulating proteins and CP. Following this, a false discovery rate (FDR) correction was applied, and colocalization analysis was conducted to provide more robust evidence supporting the links between plasma proteins and CP. Protein–protein interaction (PPI) analysis and drug evaluations were subsequently carried out. Finally, systematic MR analyses of healthy lifestyle factors and CP-associated proteins were executed to ascertain potential intervention targets for lifestyle modifications.

### 2.2. Data source and study population

The summary statistics of plasma protein genetic associations were sourced from the Pharma Proteomics Project, a precompetitive biopharmaceutical consortium that characterized the plasma proteomic profiles of 54,219 UK Biobank participants. This investigation encompassed comprehensive protein quantitative trait locus (pQTL) mapping for 2923 proteins, resulting in the identification of 14,287 primary genetic associations,^[21]^To obtain cis-pQTL, we screened pQTL based on the following criteria: pQTL exhibited genome-wide significant association (*P* < 5 × 10^−8^). Independence assumptions were satisfied (linkage disequilibrium clustering *r*^2^ < 0.001). The pQTL is cis-acting (the pQTL is within a 1000 kb window of the corresponding protein coding sequence). The pQTL is not a weak IV (*F*-statistic > 10). Echo single nucleotide polymorphisms (SNPs) and SNPs containing missing data were excluded. The genetic association data related to CP were procured from the FinnGen consortium R11, encompassing 574 cases and 449,507 controls. All participants were of European ancestry and provided informed consent (Table S1, Supplemental Digital Content, https://links.lww.com/MD/Q118). Ethical approval was granted by the relevant authorities.

## 3. Statistical analysis

### 3.1. Proteome-wide MR analysis

MR studies predict the relationship between exposure groups and outcomes by utilizing genetic variation tools based on 3 key assumptions: relevance, independence, and exclusion restriction.^[22]^ SNPs serve as IVs. The TwoSample MR package was used to execute proteome-wide MR analysis. In cases where a protein was associated with only 1 instrument variant, the Wald ratio method technique was applied to estimate associations. For proteins with 2 or more SNP alternatives, the inverse variance weighted approach with random effects was utilized to estimate and evaluate causal effects.^[23]^ Following this, 2923 plasma proteins were incorporated in the subsequent analysis. The validity of IVs was assessed utilizing *F*-statistics, with those >10 being selected, as *F*-statistics below this threshold were deemed weak IVs. After the initial MR analysis, sensitivity tests were conducted using 4 methods (weighted mode, weighted median, simple mode, and MR-Egger) to evaluate the robustness of the findings.^[24]^ Multiple testing correction was performed with the Benjamini-Hochberg method’s FDR, setting the significance threshold at FDR < 0.05. Statistical power was calculated utilizing the mRnd approach.^[25]^

### 3.2. Colocalization analysis

The colocalization analysis was conducted using the coloc R package to assess whether the observed links between circulating proteins and CP were influenced by linkage disequilibrium or whether each genomic locus harbored a single variant impacting both protein levels and CP. Bayesian colocalization analysis was employed to evaluate the support for 5 hypotheses: (H0) no causal variants for either protein or CP in the genomic locus; (H1) a single causal variant for protein alone; (H2) a single causal variant for CP alone; (H3) 2 distinct causal variants for protein and CP; (H4) a shared causal variant for both protein and CP. A posterior probability was assigned to each hypothesis. Strong evidence for colocalization between the 2 traits was considered when the posterior probability for hypothesis 4 exceeded 0.8.^[26]^ For this study, colocalization analysis was performed using separate protein cis-pQTL data and CP genome-wide association study data. To evaluate whether reverse causation might influence the results, Steiger filtering analysis was carried out using the “Two Sample MR” R package. The absence of reverse causation was indicated when the direction was “TRUE” and the *P*-value was <.05.^[27]^

### 3.3. PPIs and drug assessment

To evaluate the druggability of the detected proteins, a PPI network was developed utilizing STRING.^[28]^ The feasibility of these proteins to serve as therapeutic targets for CP was evaluated by gathering robust evidence from 3 databases: the drug-gene interaction database (DGIdb),^[29]^ ChMBE,^[30]^ and DrugBank,^[31]^ alongside druggable gene lists derived from previous research. Proteins identified as targets for any drugs or compounds in either approved or investigational stages were categorized as potential druggable targets, and detailed information on the drugs targeting these proteins was compiled.

### 3.4. Healthy lifestyle factors associated with CP-related proteins

Additionally, MR analyses were executed to explore the link between healthy lifestyle factors and CP-associated proteins, with the aim of identifying proteins whose levels could be influenced through lifestyle modifications. A sum of 11 healthy lifestyle factors (Table S2, Supplemental Digital Content, https://links.lww.com/MD/Q118, available online) were included in the analysis to assess their associations with 1 prominent CP-associated protein. The MR methodologies used were consistent with those applied in the proteome-wide MR analysis. All statistical analyses were performed using R software.

### 3.5. Heterogeneity analysis

Specifically, while single-SNP MR estimates are not subject to between-SNP heterogeneity, they may be more vulnerable to potential pleiotropy from the selected variant. To mitigate this concern, we used genome-wide significant cis-acting pQTLs, applied strict linkage disequilibrium pruning to ensure instrument independence, and complemented MR with colocalization analyses to strengthen causal inference.^[32]^

## 4. Results

### 4.1. *Protein–protein MR identified CD5 as a circulating protein associated with CP*

**Figure 1.**
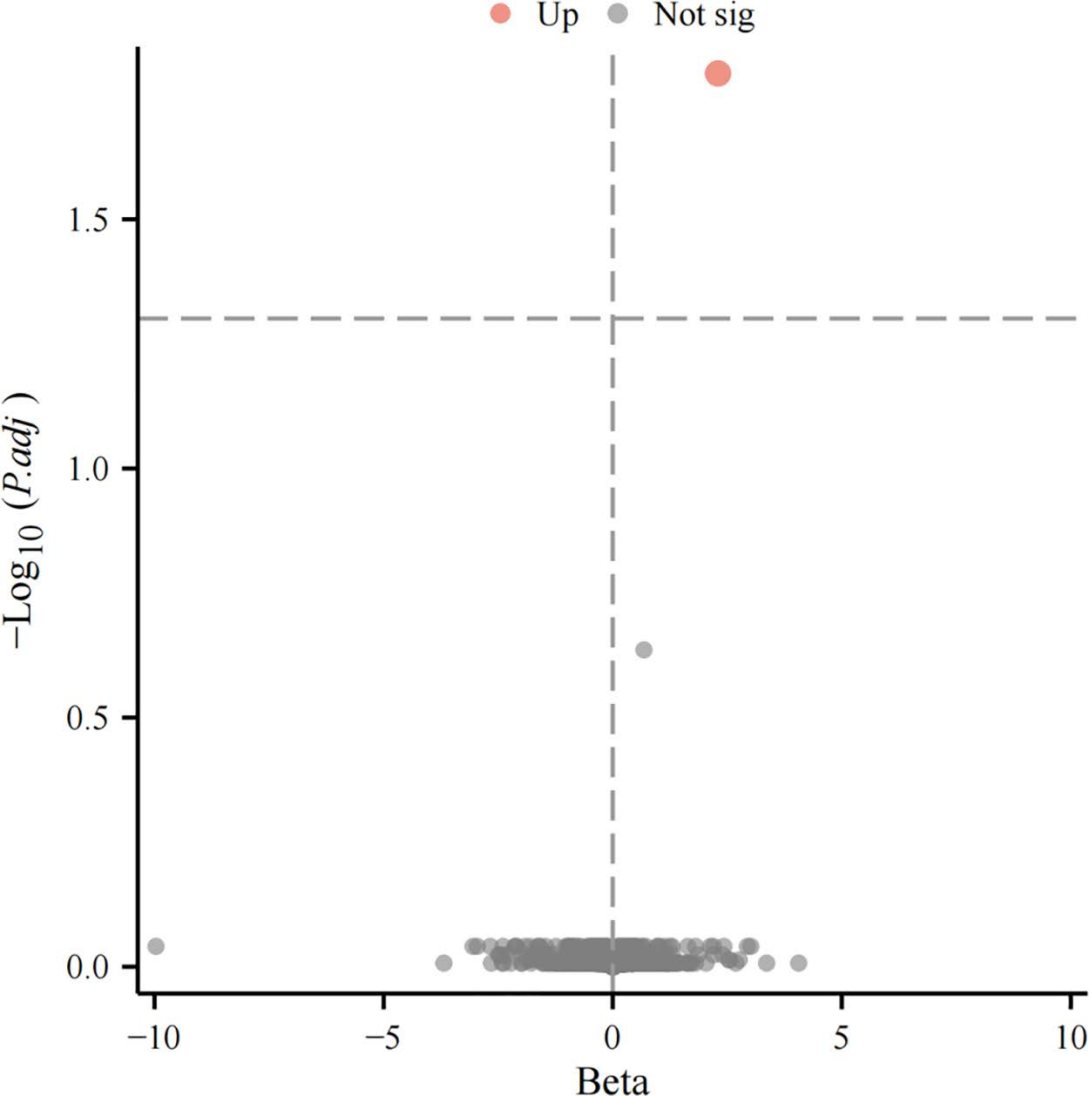
Volcano plot of proteome-wide MR analysis for CP: Only CD5 reached the significance threshold of *P* < .05 and is highlighted, suggesting a significant association with CP risk at the proteome-wide level. (The *x*-axis represents the effect size of each protein in relation to CP, and the *y*-axis indicates statistical significance. Each dot corresponds to the MR result of 1 plasma protein.) CP = cerebral palsy, MR = Mendelian randomization.

**Figure 2.**
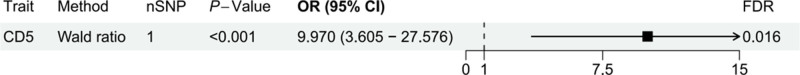
MR analysis between the CD5 on CP: CD5 may increase the risk of developing CP. CI = confidence interval, CP = cerebral palsy, FDR = false discovery rate, OR = odds ratio, MR = Mendelian randomization, SNP = single nucleotide polymorphism.

The *F*-statistics for all IVs were found to surpass 10, confirming the strength of the IVs (Table S3, Supplemental Digital Content, https://links.lww.com/MD/Q118, available online). Table S4, Supplemental Digital Content, https://links.lww.com/MD/Q118 summarizes the overall associations between plasma proteins and CP. Following the correction for multiple comparisons, a single statistically significant association was identified between a plasma protein and CP (FDR < 0.05; Fig. 1), suggesting a potential increased risk for CP: CD5 (odds ratio = 9.970, confidence interval: 3.605–27.576, *P* = 9.42 × 10⁻⁵; Fig. 2). Furthermore, no reverse causation effects were detected for any SNPs after performing Steiger filtering (Table S5, Supplemental Digital Content, https://links.lww.com/MD/Q118).

### 4.2. Verification of colocalization evidence and drug assessment

**Figure 3.**
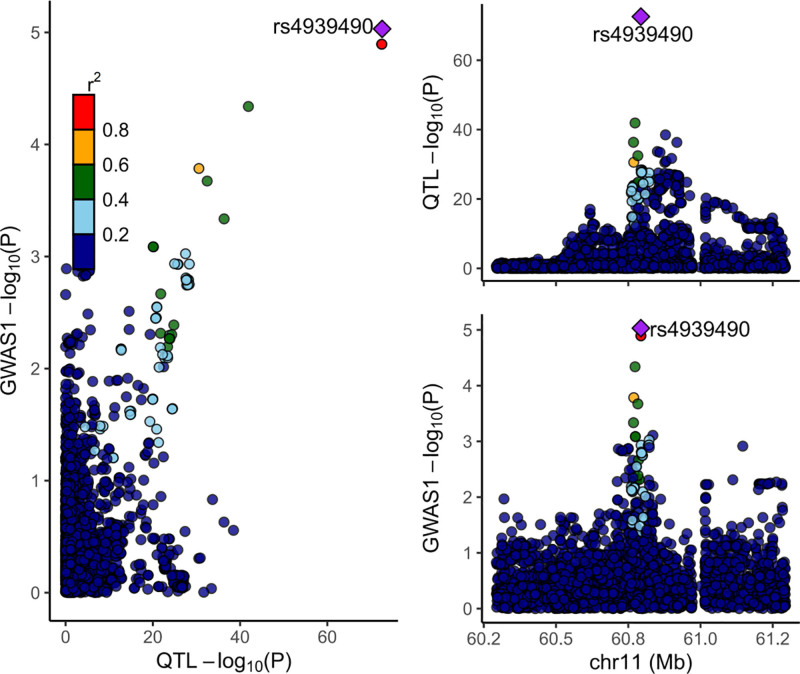
Co-localization analysis of genetic variants influencing CD5 protein levels (pQTL) with CP risk (GWAS signals): CD5 protein and CP risk exhibited consistent and strong co-localization signals across multiple genomic windows, suggesting that CD5 is a potential causal protein that may play a role in the pathogenesis of CP. CI = confidence interval, CP = cerebral palsy, GWAS = genome-wide association studies, OR = odds ratio, pQTL = protein quantitative trait locus.

**Figure 4.**
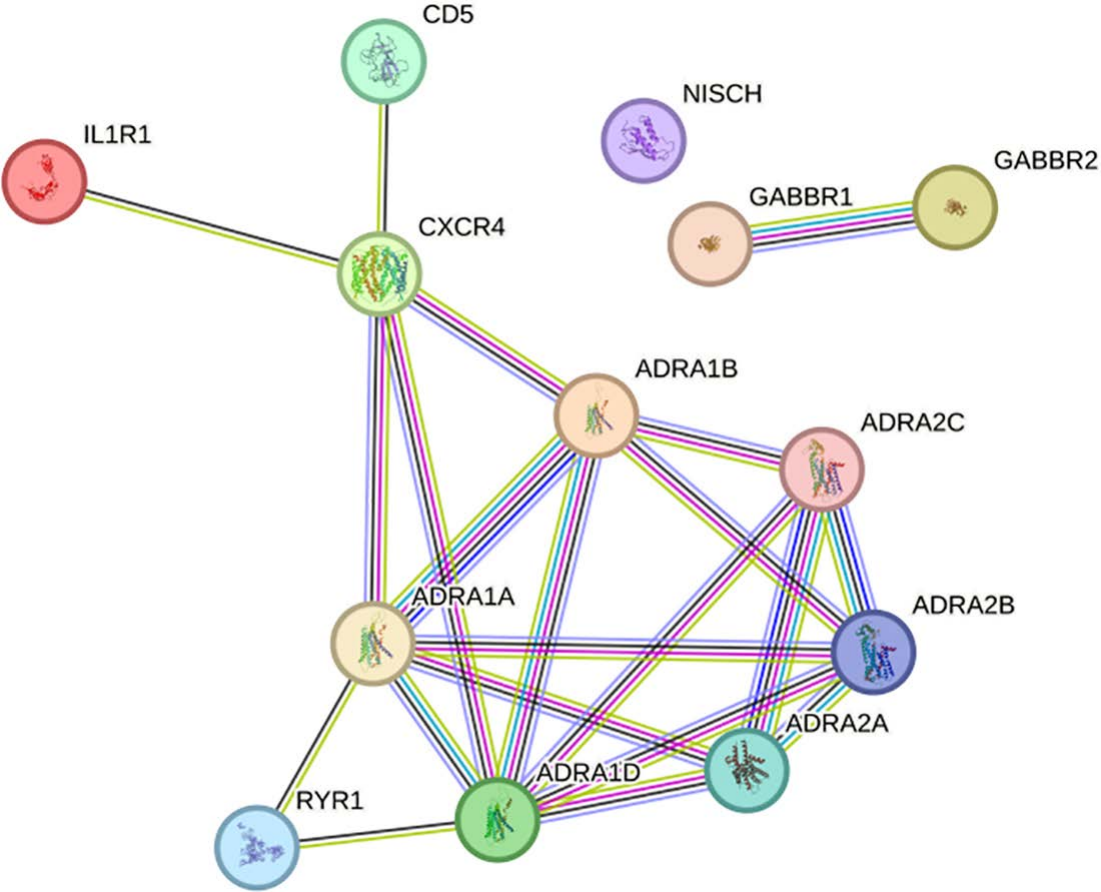
Protein–protein interaction (PPI) analysis of CD5: After PPI it was found that CXCR4 can have interactions with CD5. PPI = protein–protein interaction

After performing the colocalization analysis, substantial evidence for the colocalization of CP-related circulating protein CD5 was observed (PP4 > 0.75; Fig. 3) across different windows, suggesting probable shared causal variants between CD5 and CP (Table S6, Supplemental Digital Content, https://links.lww.com/MD/Q118). In the drug feasibility evaluation, by investigating potential drug development targets for CD5, which exhibits robust associations with CP, a promising candidate for neurological disorder drug development, protein CXCR4, was identified (Fig. 4). CXCR4 has been shown to be targeted by various therapeutic agents, including baclofen, which is used in the management of spastic CP^[33,34]^ (Table S7, Supplemental Digital Content, https://links.lww.com/MD/Q118).

### 4.3. Intervention of CP-associated proteins in healthy lifestyle

In the MR analysis involving 11 healthy lifestyle factors and CD5 protein, 3 lifestyles factors (sleep duration, smoking status: never and cheese intake) exhibited nominal associations (*P* < .05). However, the Egger test for “smoking status: never” revealed divergent directions compared to the inverse variance weighted method and the other 4 tests, resulting in its exclusion. At the same time, cheese intake should be interpreted with caution. Given the lack of prior biological evidence linking cheese consumption with CD5 protein regulation or CP pathogenesis, we consider this observation as exploratory and hypothesis-generating rather than indicative of a robust causal relationship. Future large-scale studies or replication analyses will be required to validate whether this association is genuine or reflects random variation.

**Figure 5.**
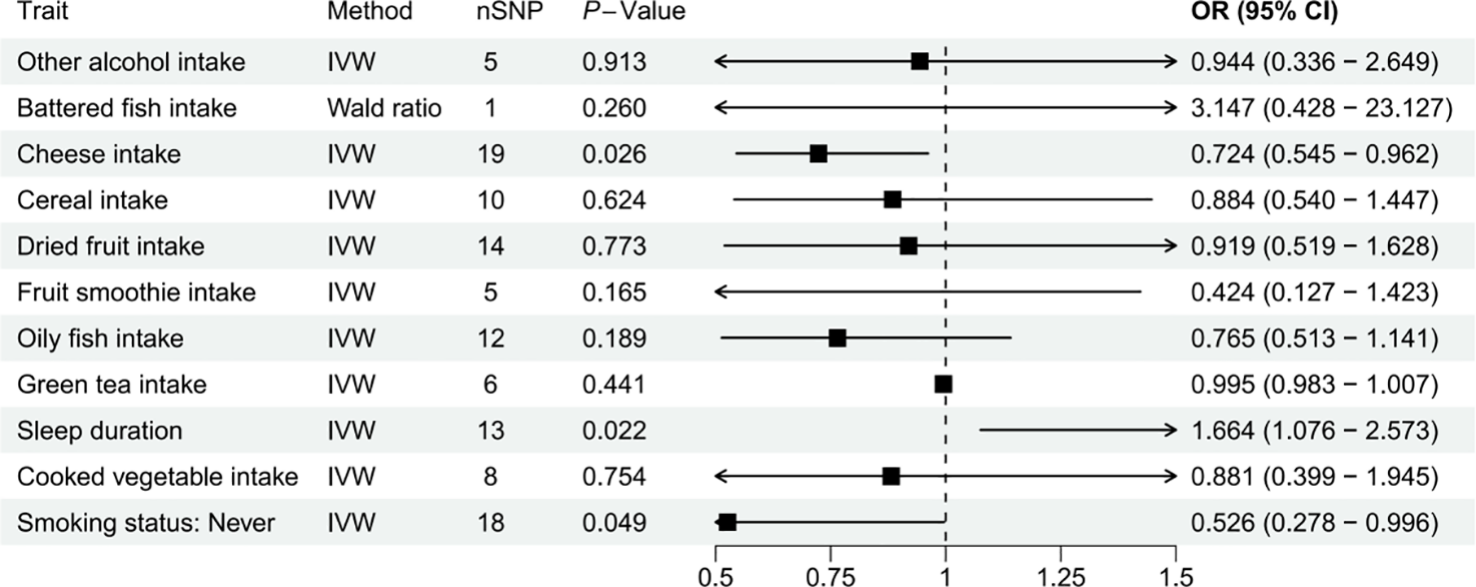
MR analysis between the lifestyles on CD5: Sleep duration, Smoking status: Never associated with CD5. CI = confidence interval, IVW = inverse variance weighted, MR = Mendelian randomization, OR = odds ratio, SNP = single nucleotide polymorphism.

After correction for multiple tests, the association between sleep duration and CD5 protein remained significant (Table S8, Supplemental Digital Content, https://links.lww.com/MD/Q118). Among the CP-associated circulating proteins, CD5 may be upregulated in response to sleep duration (Fig. 5).

## 5. Discussion

In this investigation, the causal relationships between 2923 circulating proteins and CP were thoroughly explored. One CP-related plasma protein, CD5, was identified with substantial colocalization evidence. The potential for modulating CD5 protein levels through healthy lifestyle interventions was also evaluated, revealing that CD5 plasma levels could be regulated. Despite notable progress, the molecular mechanisms underlying CP pathology remain inadequately understood,^[35]^ though a number of studies have highlighted the significant role of inflammatory factors in CP. These studies were constrained by focusing primarily on a limited set of inflammatory markers, such as interleukin families and tumor necrosis factor-alpha,^[36,37]^ without accounting for a broader spectrum of immune-related inflammatory factors. To overcome these limitations, a comprehensive examination of a larger array of circulating proteins was conducted, which led to the identification of CD5 as the protein exhibiting the strongest causal relationship. CD5, a cell surface marker found on immune cells and encoded on chromosome 11, functions both as a T-cell receptor and an immunomodulator.^[38]^ With recent advancements in inflammatory biomarker profiling, CD5’s involvement in neurological disorders has attracted increasing attention, including its role as a biomarker for neurodegenerative diseases such as Parkinson’s.^[39]^ A study examining CP children and neonatal encephalopathy (NE) revealed markedly elevated levels of various T-cells in the circulation of NE neonates, post-NE children, and CP children.^[40]^ These T-cells produced additional inflammatory factors upon in vitro stimulation, thereby exacerbating the condition.^[40]^ As a T-cell receptor, CD5 protein levels regulate T-cell content, thereby influencing inflammatory factor levels in the body. Furthermore, studies focusing on elevated inflammatory factors in the peripheral blood and cerebrospinal fluid of CP patients typically involve children aged from several months to teenage years, making it difficult to ascertain whether these elevated factors are congenital or the result of pathological conditions.^[41]^ Thus, investigating CD5, the receptor protein for T-cells that produce inflammatory factors, is of particular significance.

Through an investigation of the effects of pharmaceutical interventions and lifestyle modifications, it has been revealed that CD5 can be regulated by both medicinal treatments and healthy lifestyle practices. In terms of pharmaceutical intervention, baclofen has been employed to alleviate muscle tone disorders and pain associated with spastic CP.^[42,43]^ Furthermore, CD5 levels have been shown to be influenced by sleep duration. Studies have demonstrated that CP patients frequently suffer from poor sleep quality, difficulties in falling asleep, and insomnia,^[44]^ which are likely related to their levels of physical activity and exercise.^[45]^ The present study identified that baclofen, a drug targeting CXCR4, which has been used in spastic CP treatment, may indirectly modulate CD5-related pathways. Baclofen acts as a GABA receptor agonist, and its dystonia-relieving effects may be mediated through inhibition of the spinal reflex pathway^[46]^; however, its association with CD5 suggests a potential immunomodulatory mechanism. For example, the CXCR4 signaling pathway may influence T-cell migration to the CNS, thereby indirectly regulating CD5 levels. This hypothesis needs to be verified by pharmacological experiments. In addition, the association between sleep duration and CD5 levels provides new ideas for lifestyle intervention. Sleep deprivation has been shown to enhance pro-inflammatory cytokine secretion and disrupt the integrity of the blood-brain barrier,^[47]^ and sleep disturbances prevalent in patients with CP may exacerbate neuroinflammation by upregulating CD5. Interventions targeting sleep quality (e.g., cognitive behavioral therapy or light modulation) may be an adjunct to CP management in clinical practice. Future randomized controlled trials are needed to assess the effect of sleep interventions on the improvement of CD5 levels and CP symptoms.

After the completion of all investigations, several strengths of this study have been identified. A systematic and comprehensive approach was utilized to investigate the causal relationship between circulating proteins and CP, thereby providing a more thorough understanding of the function of circulating proteins in the pathogenesis of CP. The study incorporated carefully designed MR and colocalization analyses, which effectively mitigated concerns regarding confounding factors and reverse causation. Moreover, drug feasibility assessments and the examination of adjustable lifestyle factors for CD5 plasma protein were conducted, offering valuable insights into potential preventive and therapeutic strategies for CP through pharmaceutical interventions or lifestyle modifications. Several limitations should be noted. First, the primary focus was placed on plasma proteins. Although plasma biomarkers offer significant advantages for disease screening and diagnosis, the examination of proteins from other tissues, particularly cerebrospinal fluid, could provide more relevant insights into CP pathogenesis. Second, the sample data were exclusively procured from European populations, potentially restricting the applicability of results to other ethnic groups. Third, most links between healthy lifestyle factors and proteins were statistically significant only at the nominal level. The use of questionnaires to measure lifestyle factors in the original dataset could have introduced recall bias. Fourth, the relatively small sample size of CP patients may have influenced the identification of CP-related protein biomarkers. Fifth, although MR was implemented to minimize potential confounding factors and reverse causation, and Steiger filtering showed no evidence of reverse causation for any SNPs, proving the complete elimination of confounding factors remains challenging. Future research should involve larger, more comprehensive epidemiological studies to evaluate the link between quantified plasma protein levels and CP, alongside experimental studies to substantiate these observations. Moreover, the relatively small number of CP cases may have reduced the statistical power of Steiger filtering, thereby potentially affecting the robustness of the inferred directionality. Finally, as the identified associations are based on statistical inference, future experimental validation will be essential to confirm these causal relationships.

## 6. Conclusion

The study revealed CD5 plasma protein as a circulating biomarker with substantial causal evidence, highlighting potential protein targets for drug and lifestyle interventions, thereby offering new perspectives on the etiology, prophylaxis, and management of CP. Subsequent clinical trials are necessary to evaluate whether these discovered proteins can be successfully modulated through medications or behavioral adjustments, eventually decreasing CP susceptibility.

## Acknowledgments

We thank Bullet Edits Limited for the language editing assistance.

## Author contributions

**Conceptualization:** Chao Bai, Mingbo Hu, Junjie Wu.

**Data curation:** Mingbo Hu, Hong Zhao, Junjie Wu, Xinping Luan, Song-hai Biedelehan.

**Formal analysis:** Chao Bai, Jingxuan Xu.

**Funding acquisition:** Xinping Luan, Song-hai Biedelehan.

**Investigation:** Hong Zhao.

**Methodology:** Jingxuan Xu.

**Software:** Song-hai Biedelehan.

**Validation:** Xinping Luan.

**Visualization:** Xinping Luan.

**Writing – original draft:** Chao Bai, Mingbo Hu, Hong Zhao, Junjie Wu.

**Writing – review & editing:** Jingxuan Xu, Xinping Luan, Song-hai Biedelehan.

## Supplementary Material


